# Effect of immune-modulating metronomic capecitabine as an adjuvant therapy in locoregionally advanced nasopharyngeal carcinoma

**DOI:** 10.1186/s12865-024-00621-3

**Published:** 2024-05-06

**Authors:** Qianyong He, Xiuling Luo, Lina Liu, Chaofen Zhao, Zhuoling Li, Feng Jin

**Affiliations:** 1 Department of Oncology, the Affiliated Hospital of Guizhou Medical University, 28 Guiyi Street, Guiyang, Guizhou 550004 P.R. China; 2 Department of Oncology, the Affiliated Cancer Hospital of Guizhou Medical University, Guiyang, Guizhou 550001 P.R. China; 3 School of Clinical Medicine, Guizhou Medical University, Guiyang, Guizhou 550004 P.R. China

**Keywords:** Adjuvant therapy, Immune function, Metronomic capecitabine, Nasopharyngeal cancer, Survival rate

## Abstract

**Introduction:**

Metronomic capecitabine used as an adjuvant therapy improves survival in patients with locoregionally advanced nasopharyngeal carcinoma (LA-NPC). This therapeutic approach may also contribute to improving immune function, consequently enhancing overall therapeutic efficacy.

**Aim:**

We aimed to evaluate the effect of metronomic capecitabine as adjuvant therapy on immune function and survival in cases of LA-NPC.

**Subjects and methods:**

28 patients with LA-NPC were enrolled in the study and equally assigned to two groups of 14 each: experimental and control group. The experimental group received induction chemotherapy + concurrent chemotherapy + adjuvant chemotherapy as well as oral capecitabine at a dose of 650 mg/m² of body surface area twice daily for 1 year, with the option to discontinue in case of intolerance. The control group did not receive additional chemotherapy or targeted drugs after the induction chemotherapy + concurrent chemoradiotherapy; however, they were followed up regularly. Changes in immune function and survival were compared between the two groups.

**Results:**

The median follow-up time was 43.5 months. One year after adjuvant chemotherapy, the experimental group showed higher levels of CD8 + cells, CD28 + CD8 + cells, and activated CD8 + cells compared to the control group (*P* < 0.05). The CD4/CD8 ratio and proportion of monocyte-derived dendritic cells were also higher in the experimental group than in the control group, but the difference was not statistically significant (*P* ≥ 0.05). Comparisons of 3-year overall survival, local-regional recurrence-free survival, progression-free survival, and distant metastasis-free survival between the two groups showed percentages of 92.9% vs. 78.6%, 92.9% vs. 92.9%, 78.6% vs. 71.4%, and 85.7% vs. 0.78 0.6% respectively, but these differences were not significant (*P* > 0 0.05 ).

**Conclusion:**

Metronomic capecitabine chemotherapy was observed to induce an immunomodulatory effect in LA-NPC.

**Trial registration:**

NCT02958111, date of registration 04-11-2016.

**Supplementary Information:**

The online version contains supplementary material available at 10.1186/s12865-024-00621-3.

## Introduction

In Southeast China, there is a high prevalence of nasopharyngeal carcinoma (NPC) with a distinct regional distribution [1]. Globally, more than 130,000 new cases of nasopharyngeal cancer were reported in 2021, leading to approximately 80,000 deaths [2]. A multicenter phase 3 clinical trial conducted at our center on this subject has demonstrated that metronomic capecitabine used as adjuvant therapy, improves failure-free survival in patients with high-risk locoregionally advanced NPC (LA-NPC). This trial is registered with ClinicalTrials.gov under NCT02958111 [3]. Studies on glioblastoma have shown that low-dose capecitabine metronomic chemotherapy acts as an immunomodulator, thereby reducing the number of myeloid-derived suppressor cells in patients [4]. In a retrospective analysis conducted by Shen [5], 677 patients with nonmetastatic NPC were included [4]. Patients with higher CD4(+) T cell counts showed superior 5-year progression-free survival (PFS) (83% vs. 74.2%, *P* = 0.015) and improved 5-year distant metastasis-free survival (DMFS) (95.8% vs. 86.7%, *P* = 0.001) compared to those with low CD4(+) T cell counts, respectively. Multivariate analysis results indicated that CD4(+) T cells were distinct predictive factors for both PFS and DMFS. During one month of chemoradiotherapy, the percentages of CD4(+) T cells decreased, while the percentages of CD8(+) T cells and the CD4/CD8 ratio increased. In addition, a study that matched clinical factors with the lymphocyte subsets of 220 patients with NPC found that younger patients had higher percentages of CD3 + and CD3 + CD8 + cells compared to older patients. Higher percentages of CD3 + CD8 + or lower percentages of CD3 − CD56 + cells were associated with better overall survival (OS) rates. The results of multivariate survival studies identified the percentage of CD3 + CD8 + cells as a separate prognostic factor [6]. Studies have confirmed that metronomic chemotherapy can exert an immunomodulatory effect in lung cancer [7], colon cancer [8], and recurrent ovarian cancer [9]. Therefore, we hypothesized that capecitabine metronomic chemotherapy improves failure-free survival in patients with high-risk LA-NPC and may also contribute to improved immune function. To investigate the impact of adjuvant capecitabine metronomic treatment on survival and immunological function in patients with LA-NPC, we analyzed the lymphoid subsets of 28 patients.

## Subjects and methods

### Enrollment criteria

From November 2017 to October 2018, a total of 28 patients with NPC were enrolled in the study at the Affiliated Cancer Hospital of Guizhou Medical University. The enrollment criteria include the following: (1) patients newly diagnosed with NPC; (2) aged 18–70 years; (3) clinical stage III–IVa (American Joint Committee on Cancer [AJCC] staging manual version 8); (4) absence of distant metastasis; (5) Karnofsky Performance Status score of ≥ 70 with no serious underlying diseases; and (6) patients undergoing intensity-modulated radiation therapy (IMRT). Patients who did not meet the above criteria were excluded. A total of 14 patients with NPC were assigned to the experimental group. The treatment plan included induction chemotherapy + concurrent chemotherapy + adjuvant chemotherapy. A control group was also formed, consisting of 14 patients who received induction chemotherapy and concurrent chemotherapy. The trial used receipt of adjuvant chemotherapy (yes or no) for stratification. Within 12–16 weeks after chemoradiotherapy, patients were assigned (1:1; block size four) to receive either clinical observation only (control group) or adjuvant metronomic capecitabine (experimental group). The study was conducted according to the principles of the Declaration of Helsinki, and the results were reported by CONSORT. The study was registered at ClinicalTrials.gov (No. NCT02958111, date of registration 04-11-2016) and approved by the Ethics Committee of Affiliated Cancer Hospital of Guizhou Medical University (Ethics No. SL-201,701,008).

### Treatment protocol

The treatment protocol was as follows: Induction chemotherapy: docetaxel (75 mg/m^2^, intravenous injection on day 1); cisplatin (75 mg/m^2^, intravenous injection on day 1); 5-fluorouracil (750 mg/m^2^ per day, civ, days 1–5), three cycles, 21 days/cycle. Radiotherapy began 3–4 weeks after the completion of induction chemotherapy. Concurrent chemotherapy: concurrent single-agent cisplatin chemotherapy for two cycles, 21 days/cycle: cisplatin (100 mg/m^2^, civ, day 1). Radiotherapy: All patients were given 6MV-X radiation via Intensity Modulated Radiotherapy(IMRT) while in a supine position, with the head, neck, and shoulder frame in a fixed position. Specific radiation dose: T3-4 GTVnx (when assessing the gross tumor volume, the primary tumor sites visualized by imaging and endoscopy were included) = 72.6 Gy/33f, T1-2 GTVnx = 69.96 Gy/33f, PGTVnx (GTVnx + 5 mm) = 69.96 Gy/33f, GTVnd (including only the sites of metastatic lymph nodes that could be determined by imaging and palpation) = 69.96 Gy/33f, CTV1 (clinical target volume, PGTVnx + surrounding high-risk area + upper cervical lymph node drainage area); PTV1 (plan target volume, CTV1 + 3 mm) = 60.06 Gy/33f; CTV2 (the drainage area of the middle and lower cervical lymph nodes); PTV2 (CTV2 + 3 mm) = 50.96 Gy/28f, spread over 7–8 weeks. Adjuvant chemotherapy: capecitabine 650 mg/m^2^, administered orally twice daily for 1 year as maintenance treatment, with the option to discontinue if intolerable due to toxic effects. Treatment protocol for the control group: no additional chemotherapy or targeted drugs after induction chemotherapy + concurrent chemoradiotherapy, with regular follow-ups.

### Detection of lymphocyte subsets

Peripheral blood lymphocyte subsets were detected using six-color fluorescence labeled flow cytometry with monoclonal fluorescent antibodies provided by Becton, Dickinson, and Company (USA). A BD FACScont flow cytometer was used to automatically obtain and analyze 50,000 cells from approximately 2 mL of peripheral blood collected from each patient. A 20-µL antibody mixture was prepared and incubated in the dark at room temperature for 15 min. Subsequently, 450-µL hemolysin was added, and the mixture was allowed to react in the dark at room temperature for 15 min. The measured values were analyzed using Random software. Peripheral blood lymphocyte subsets were detected in both groups 1 year after the completion of radiotherapy and capecitabine treatment.

### Detection of dendritic cell (DC) subsets

For fluorescent antibody staining of whole blood samples, four experimental tubes were used; 10-µL Lin-1, 5-µL CD123, 5-µL CD11c, and 5-µL HLA-DR were added to each tube. Additionally, four control tubes were prepared, with 5 µL each of Lin-1, Mouse IgG1, Mouse IgG2 a, and HLA-DR added to each tube. Then, 100 µL of whole blood was added to each tube, mixed with shaking, and incubated in the dark for 15 min. An additional 2 mL of FACS hemolysin was added to each tube to dissolve erythrocytes, and the cells were allowed to act for 5–8 min. The samples were centrifuged at 1,500 rpm for 5 min, and the supernatant was discarded. The mixture was mixed with shaking, and 1 mL phosphate-buffered saline (PBS) wash was added to each sample. The samples were centrifuged at 1,200 rpm for 5 min, and the supernatant was discarded. The mixture was mixed with shaking, and 300 µL of PBS was added to it, followed by storage in the dark at 2–8 °C and separation within 24 h. A BD FACScont flow cytometer was used in this study. Using CELLQuest software, 100 cells were obtained, and the cells positive for both CD11c and anti-HLA-DR were identified as CD11c + DCs. Cells positive for both CD123 and anti-HLA-DR were CD123 + DCs. Plasmacytoid DCs (PDCs) were defined as Lin-HLA-DR + CD123c+, whereas monocyte-derived dendritic cells (MDCs) were defined as Lin-HLA-DR + CD11c+. DCs were detected in both groups 1 year after the completion of radiotherapy and capecitabine treatment.

### Outcomes of lymphocyte subset and DC examination, along with clinical efficacy evaluation

Main study objective: to observe changes in lymphocyte count and the proportion of CD4 + cells, CD8 + cells, CD4+/CD8 + ratio, PDCs, and MDCs in the two groups of patients before and after adjuvant treatment in the experimental group. The secondary observation index was the survival rate. OS rate: the time from the study’s initiation to death due to any cause. Local-regional recurrence-free survival (LRRFS) rate: the survival rate from the study’s initiation to the occurrence of local-regional recurrence. DMFS rate: the survival rate from the beginning of the study to the time of distant metastasis. Progression-free survival (PFS): the survival rate from the study’s initiation to the time of tumor progression or death.

### Follow-up

Follow-ups were conducted through patient reviews and telephone interviews. The follow-up schedule included visits once every 3 months within 2 years after treatment, and once every 6 months after the third year. The follow-up included an assessment of the patient’s general condition; routine physical examination; routine blood work; liver and kidney function analysis; Epstein-Barr virus DNA testing; electronic nasopharyngoscopy; magnetic resonance imaging of the nasopharynx and neck, chest, and upper abdomen; computed tomography; whole-body bone scan; and evaluation of lymphocyte subsets and DC subsets.

### Statistical analysis

Statistical analysis was performed using SPSS 26.0 software. Measurement data and indicators conforming to normal distributions were presented as the mean ± standard deviation (*x* ± SD), whereas indicators that did not meet normal distributions were presented as the median and interquartile range. Count data were expressed as a percentage (%). The changing trends of lymphocyte and DC subsets in the peripheral blood were observed, and analysis of variance was applied to normally distributed datasets to assess for congruency, whereas the Friedman M test was used for comparing multiple related samples. Survival probabilities were calculated using the Kaplan–Meier method, and survival curves were compared using the log-rank test.

## Results

Between November 2017 and October 2018, 28 patients underwent screening and were enrolled in this study. They were randomly assigned to the experimental group (*n* = 14) or the control group (*n* = 14). Detailed clinical information, including sex, age, and clinical stage, is presented in Table 1.

**Table 1:** Clinical characteristics of 28 patients with LA-NPC

Characteristics	Control group	Experimental group	*p*
N	14	14	
Sex, n (%)			0.678
Female	3 (21.43%)	5 (35.71%)	
Male	11 (78.57%)	9 (64.29%)	
Age (years), mean ± SD	47.79 ± 7.82	42.71 ± 11.19	0.176
Histologic subtypes, n (%)			
Undifferentiated carcinoma	14(100%)	14(100%)	1.000
T stage, n (%)			0.042
T2	2 (14.29%)	0 (0%)	
T3	1 (7.14%)	6 (42.86%)	
T4	11 (78.57%)	8 (57.14%)	
N stage, n (%)			0.775
N1	4 (28.57%)	3 (21.43%)	
N2	8 (57.14%)	7 (50.0%)	
N3	2 (14.29%)	4 (28.57%)	
Clinical stage, n (%)			1.000
III	2 (14.29%)	2 (14.29%)	
IVa	12 (85.71%)	12 (85.71%)	
EBV-DNA copies/ml after radiotherapy			0.549
Positive	3(21.43%)	4(28.57%)	
Negative	11 (78.57%)	10(71.43%)	

### Effect on immune function

**Fig. 1:**
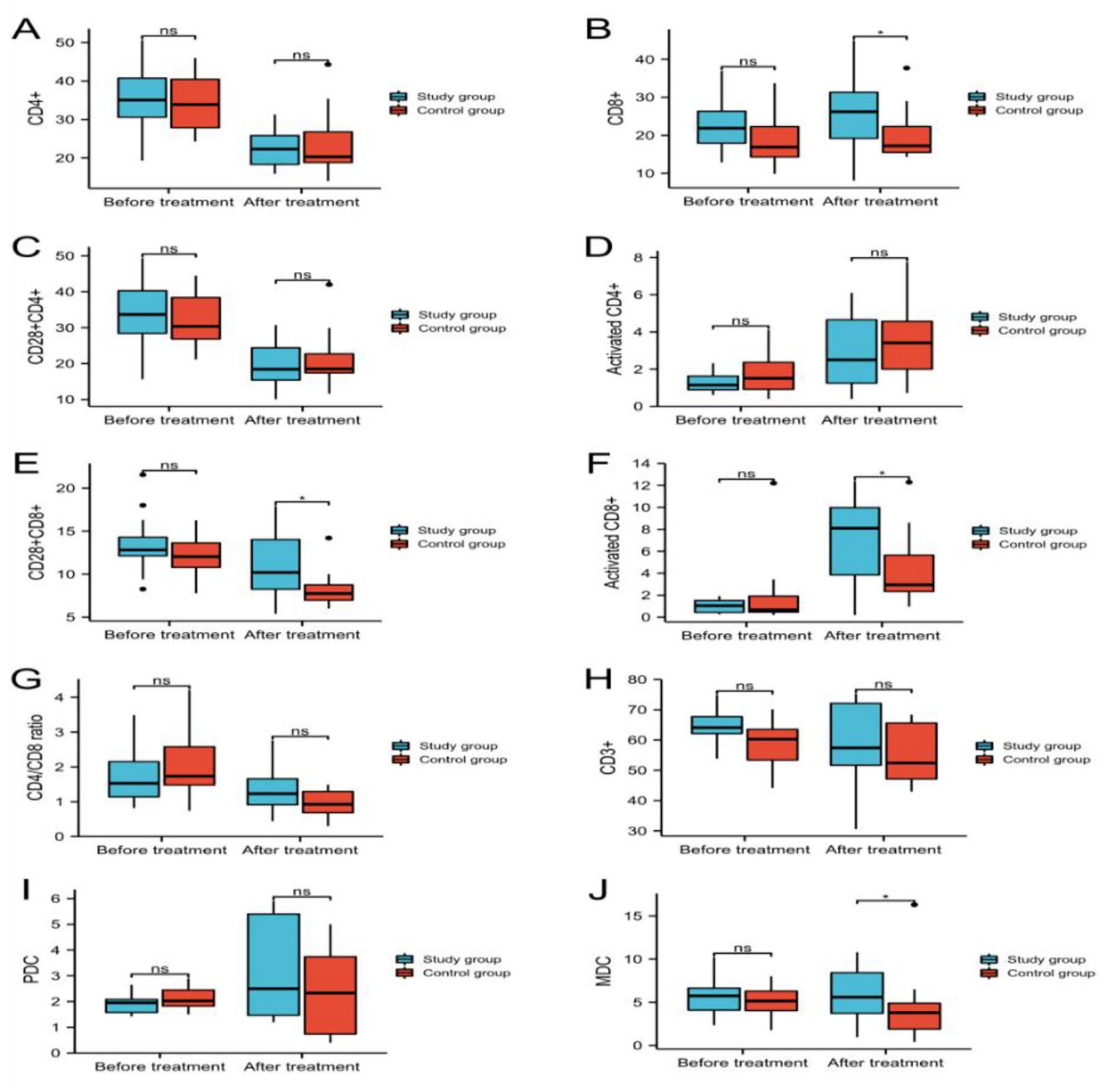
Comparison of CD4+(**A**), CD8+(**B**), CD28 + CD4+(**C**), Activated CD4+(**D**), CD28 + CD8+(**E**), Activated CD8+(**F**), CD4+/CD8 + ratio(**G**), CD3+(**H**), PDCs (**I**), MDCs (**J**)before and after treatment (ns, *p* ≥ 0.05; *P < 0.05; **P < 0.01; ***P < 0.001)

The difference in lymphocyte count, CD4 + cells, CD8 + cells, CD28 + CD4 + cells, activated CD4 + cells, CD28 + CD8 + cells, activated CD8 + cells, the ratio of CD4+/CD8+, CD3 + cells, PDCs and MDCs between the experimental and control groups before induction chemotherapy was not statistically significant (*P* > 0.05) (Table 2; Fig. 1).

After receiving post-adjuvant chemotherapy, the experimental group showed statistically significant increases in CD8 + cells, CD28 + CD8 + cells, and activated CD8 + cells compared to the control group. The difference was statistically significant (*P* < 0.05). Although CD4 + cells, CD28 + CD4 + cells, activated CD4 + cells, CD3 + cells, and PDCs also exhibited higher levels in the experimental group than in the control group, the differences were not statistically significant (*P* > 0.05). The CD4/CD8 ratio was higher in the experimental group than in the control group, indicating a trend toward immune function enhancement (*P* = 0.06) (Table 3; Fig. 1).

In the experimental group, lymphocyte counts, lymphocyte subsets, and dendritic cells before and after treatment. Through comparison, it was found that CD4 + cells, CD28 + CD4 + cells, and CD4/CD8 ratio significantly decreased after capecitabine treatment compared to before treatment, and the difference was statistically significant (*P*<0.05) (Table 4).

**Table 2:** Median values (interquartile spacing) for comparison between lymphocyte count, lymphocyte subsets, and dendritic cells before the treatment

Group	Lymphocyte count	CD4+	CD8+	CD28 + CD4+	Activated CD4+	CD28 + CD8+	Activated CD8+	CD4/CD8 ratio	CD3+	PDCs	MDCs
Experimental group	1.70 × 10^9^(1.33–2.04 × 10^9^)	35.05(19.34–50.60)	21.89(12.90-36.94)	33.66(15.61–49.37)	1.15(0.61–2.31)	12.81(8.27–21.55)	1.05(0.27–1.93)	1.53(0.82–3.49)	64.10(53.85–74.83)	2.50(1.20–5.90)	5.75(2.34–10.20)
Control group	1.91 × 10^9^(1.42–2.39 × 10^9^)	33.91(24.25–45.99)	16.92(9.88–33.70)	30.35(21.17–44.46)	1.51(0.40–4.10)	12.04(7.78–16.26)	0.67(0.20–12.20)	1.74(0.74–4.21)	60.26(44.14–70.18)	2.33(0.40–2.90)	5.15(1.78-8.00)
Z	1.199	-0.58	-3.38	-1.60	0.40	-1.13	-0.02	0.86	-5.12	0.18	-0.39
*P*	0.376	0.84	0.19	0.64	0.21	0.29	1.00	0.40	0.05	0.24	0.64

**Table 3:** Median values (interquartile spacing) of the two lymphocyte counts, lymphocyte subsets, and dendritic cells after treatment

Group	Lymphocyte count	CD4+	CD8+	CD28 + CD4+	Activated CD4+	CD28 + CD8+	Activated CD8+	CD4/CD8 ratio	CD3+	PDCs	MDCs
Experimental group	1.48 × 10^9^(1.06–1.86 × 10^9^)	22.30(15.90–31.30)	26.22(8.10–44.90)	18.40(10.10–30.70)	2.50(0.40–6.10)	10.20(5.40–17.90)	8.10(0.20–12.40)	1.23(0.44–2.76)	57.40(30.60–75.30)	2.50(1.20–5.90)	5.60(0.97–10.79)
Control group	1.85 × 10^9^(1.30–1.98 × 10^9^)	20.31(14.00-44.30)	17.25(14.30–37.70)	18.50(11.60–42.00)	3.42(0.72–7.76)	7.75(6.00-14.20)	2.95(0.96–12.29)	0.93(0.30–1.49)	52.39(43.00-68.36)	2.33(0.40-5.00)	3.80(0.40–16.30)
Z	0.298	0.51	-4.86	0.90	0.55	-2.19	-3.05	-2.01	-4.81	-1.00	-2.30
*P*	0.185	0.84	0.03	0.73	0.46	0.03	0.04	0.06	0.18	0.13	0.05

**Table 4:** Comparison of lymphocyte count, lymphocyte subsets, and dendritic cells before and after treatment with carbidopa in the experimental group(x ± s)

Group	Lymphocyte count	CD4+	CD8+	CD28 + CD4+	Activated CD4+	CD28 + CD8+	Activated CD8+	CD4/CD8 ratio	CD3+	PDCs	MDCs
Before treatment	1.70 × 10^9^±0.14 × 10^9^	33.08 ± 3.73	21.97 ± 1.81	31.78 ± 4.25	3.73 ± 0.79	12.52 ± 0.85	3.96 ± 0.88	1.53 ± 0.20	60.91 ± 3.75	2.14 ± 0.040	8.36 ± 2.21
After treatment	1.48 × 10^9^±0.15 × 10^9^	23.11 ± 1.71	24.22 ± 3.13	20.20 ± 2.04	3.14 ± 0.64	11.38 ± 1.12	5.85 ± 1.50	1.14 ± 0.17	52.04 ± 3.64	2.20 ± 0.54	5.79 ± 1.18
t	1.171	3.48	-1.049	3.725	0.694	1.080	-1.281	3.34	1.992	-1.121	1.78
*P*	0.263	0.006	0.319	0.004	0.504	0.306	0.229	0.007	0.074	0.906	0.263

### Disease outcome and survival

**Fig. 2:**
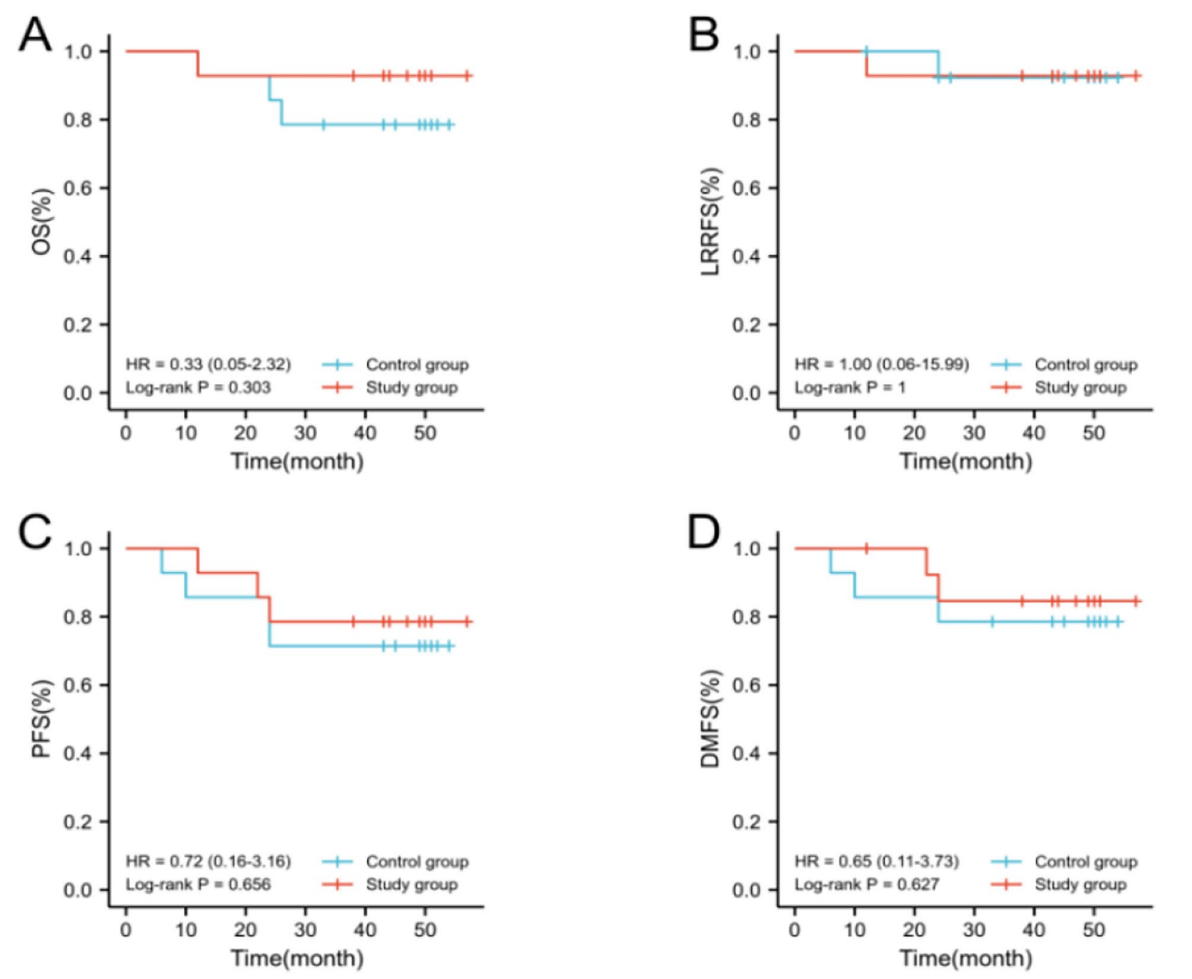
Survival comparison of metronomic capecitabine adjuvant chemotherapy in patients with LA-NPC (A: OS, B: LRRFS, C: PFS, D: DMFS)

As of August 2022, the median follow-up duration for all patients was 43.5 months (ranging from 43 to 51). All patients in both the experimental and control groups completed three cycles of induction chemotherapy and two cycles of concurrent cisplatin protocol. Additionally, all patients in the experimental group underwent adjuvant chemotherapy following the capecitabine metronomic protocol. One patient in the experimental group experienced a relapse and died due to massive nasopharyngeal hemorrhage, while two patients developed distant metastasis. In the control group, one patient had a recurrence and three patients developed distant metastasis leading to death. The 3-year OS of the patients in the experimental group and control group was 92.9% and 78.6%, respectively (Hazard ratio(HR) = 0.33,95% confidence interval(CI) 0.05–2.32, *P* = 0.303). The 3-year LRRFS was both measured at 92.9% (HR = 1.00,95%CI 0.06–15.99, *P* = 1.000). The PFS at 3 years was recorded as 78.6% for the experimental group and as 71.4% for the control group (HR = 0.72,95%CI 0.16–3.16, *P* = 0.656), while the DMFS at 3 years was found to be at a rate of 85.7% for the experimental group and at a rate of 78.% for the control group(HR = 0.65,95%CI 0.11–3.73, *P* = 0.627)(Fig. 2A, B, C, and D).

## Discussion

The primary form of treatment for NPC is radiotherapy. Initially, a combination of advanced local comprehensive treatments with concomitant radiochemotherapy and either adjuvant chemotherapy or radical radiotherapy is employed. Although studies have demonstrated that adjuvant chemotherapy with a platinum and fluorouracil regimen does not adequately lower the likelihood of treatment failure, adjuvant chemotherapy with a cisplatin and fluorouracil regimen has long been established as the standard adjuvant chemotherapy program [10]. Exploring a “high efficiency” mode is crucial for enhancing the effectiveness of NPC treatment. Pandey [11] retrospectively analyzed the survival times of patients with locally advanced oral cancers and compared the OS of patients without metronomic chemotherapy to those with metronomic chemotherapy (26 months vs. 14 months, *P* = 0.04). The study found that the survival times were significantly prolonged among the patients who underwent metronomic chemotherapy. Another trial involving patients with stage IV nonmetastatic oral cancer revealed that those who received metronomic chemotherapy had 3-year disease-free survival rates of 53.05% compared to 35.41% in the control group (*P* = 0.011), and 3-year OS rates of 74.96% compared to 48.47% in the control group (*P* = 0.001) respectively [12]. According to the findings of a multicenter phase 3 clinical trial, metronomic capecitabine as an adjuvant medication improved failure-free survival in patients with high-risk LA-NPC [3]. Additionally, metronomic chemotherapy given to patients with NPC improved their OS. Therefore, metronomic chemotherapy offers a unique mode of delivery for adjuvant chemotherapy after concurrent chemoradiotherapy in LA-NPC.

According to our findings, the proportions of CD8 + cells, CD28 + CD8 + cells, and activated CD8 + cells were increased compared to those in the control group. Yuan [13] reported that, in addition to its immune-activation effects, metronomic chemotherapy with the fluorouracil drug inhibited the proliferation of gastric cancer cells both internally and externally by anti-tumor angiogenesis. Another study [7] has suggested that metronomic capecitabine chemotherapy reduces the levels of circulating myeloid-derived suppressor cells and improves the immune microenvironment. Lymphocyte subsets are an important component of the body’s immune defense, playing a key role in immune surveillance and anti-tumor immunity. Past studies have found that T-lymphocyte-mediated cellular immunity can kill and clear tumor cells, as well as inhibit the growth of tumor cells [14, 15]. The T-lymphocyte subsets include CD3 + cells, CD4 + cells, CD8 + cells, CD4/CD8 ratio, and CD4 + CD25 + cells, as indicated.Among them, CD3 + cells are mainly present on the surfaces of mature T-lymphocytes and can participate in T-cell conduction. Its proportion indicates the level of T lymphocytes, and decreased expression indicates a weakened immune response [16]. Studies have shown that high levels of CD3 + cells cells are associated with a favorable OS [17]. CD8 + T-activatable effector cells are cytotoxic T cells [18], and previous studies have detected CD8 + cells in NPC microenvironments, which can predict treatment effectiveness [19]. CD4 + cells and CD8 + cells are important components of T lymphocyte immune regulation. CD4 + cells can induce B-cell differentiation, while CD8 + cells can inhibit B-cell antibody synthesis and the proliferation of T lymphocytes. A reduced CD4/CD8 ratio may disrupt immune balance and lower ratios are an independent prognostic factor for DMFS [20]. DCs play a crucial role in tumor microenvironments, with previous studies indicating that high expression of DCs is a significant marker for good prognosis in head and neck cancers [21]. Our results align with those of Laurent et al. [22]. CD4 + cells and CD8 + cells are important components of T lymphocyte immune regulation; CD4 + cells can induce B-cell differentiation; and CD8 + cells can inhibit B-cell antibody synthesis and the proliferation of T lymphocytes. The CD4/CD8 ratio can reduce the immune balance and lower CD4/CD8 ratios and is an independent prognostic factor for DMFS [20]. DCs are important cells in tumor microenvironments, and past studies have shown that a high expression of DCs is an important marker of good prognosis in head and neck cancers [21]. Our results are in agreement with those of Laurent et al. [22]. In this study, we selected the cut-off point of 1 year as the end of capecitabine oral administration, excluding inflammatory manifestations or infections affecting lymphocyte subsets caused by acute mucositis, dermatitis, or toxic or other side effects of radiotherapy. In summary, metronomic chemotherapy kills tumor cells while relieving immune suppression in the body, thereby improving the regulatory functions of lymphocytes and DCs.

This study included 28 individuals with T4, N1-3, or any T, N2-3 LA-NPC. The control.

the group did not receive any treatment following induction chemotherapy with concurrent chemoradiotherapy; the experimental group received induction chemotherapy along with concurrent chemoradiotherapy and metronomic capecitabine chemotherapy. The findings showed that the 3-year OS, LRRFS, PFS, and DMFS scores of the experimental group were higher than those of the control group; however, these differences were not statistically significant (*P* > 0.05). Chen et al. [3] demonstrated in their study that the 3-year OS rate of the metronomic capecitabine chemotherapy group was significantly higher than that of the standard treatment group, at 93.3% (95% CI 89.7–97.1) vs. 88.6% (95% CI 84.2–93.2); the 3-year DMFS rate was 89.4% (95% CI 85.1–94.0) vs. 82.1% (95% CI 76.8–87.8); and the LRRFS rate was92 0.6%(95% CI88.7-96.6) vs. 87.8%(95% CI 83.1–92.7). According to these findings, adjuvant metronomic capecitabine treatment prolongs survival in patients with LA-NPC. Although our data indicated that patients receiving adjuvant metronomic capecitabine therapy tended to have a longer OS, LRRFS, PFS, and DMFS compared with those of the control group. We did not achieve significant survival results, potentially due to the small patient sample and the inclusion of more N3 patients in the experimental group. Additionally, the control group included 2 patients with T2 stage. Another reason was that after chemotherapy with capecitabine, we found that the levels of CD8 + cells, CD28 + CD8 + cells, and activated CD8 + cells increased. Zhu’s [6] study showed that the percentage of CD8 + cells in peripheral blood increased after radiotherapy and could achieve better OS. A retrospective cohort study indicated that the percentage of CD4 + cells and the CD4/CD8 ratio decreased in peripheral blood after radiotherapy, but multivariate analysis revealed that CD4 + cells and the CD4/CD8 ratio were prognostic factors [23]. In our comparative experimental group, we also found a decrease in lymphocyte subsets and dendritic cells after treatment, consistent with previous research results: specifically, a decrease in CD4 + cells, CD28 + CD4 + cells, and the CD4/CD8 ratio after treatment.

Although the study found some promising results, there are also some limitations. Firstly, a.

single center was used and the patient sample size was small. Secondly, these results should be stratified by T, N, and clinical stage to understand if infiltration of the tumor in local lymph nodes results in different modulation of the immune system after treatment and if the modulation of immune cells after treatment is also affected by clinical stage (stage III vs. stage IVa). However, Table 1 showed the T, N stage, and total stage with several patients in each group which restricted stratified analysis. Finally, the follow-up time was less than 5 years. Therefore, our next study will further expand the sample size, extend follow-up time and closely monitor the immune function of patients.

## Conclusion

Our findings demonstrated higher levels of CD8 + cells, CD28 + CD8 + cells, and activated CD8 + cells in patients with LA-NPC 1 year after adjuvant metronomic capecitabine treatment compared to those in the control group. These increased levels tended to extend OS, LRRFS, PFS, and DMFS in these patients compared to those in the control group. Despite some limitations, our findings highlight that the study confirmed the impact of metronomic chemotherapy on immune functions in LA-NPC.

### Electronic supplementary material

Below is the link to the electronic supplementary material.

Supplementary Material 1

Supplementary Material 2

Supplementary Material 3

## Data Availability

The data that support the findings of this study are available when requested. from the corresponding author, [Feng Jin], upon reasonable request.
