# Supplementary material for: Effect of immune-modulating metronomic capecitabine as an adjuvant therapy in locoregionally advanced nasopharyngeal carcinoma
Source: BMC Immunol. 2024 May 6;25:28. doi: 10.1186/s12865-024-00621-3 (PMC11071185; doi:10.1186/s12865-024-00621-3)
Supplement: Supplementary file 1 — Supplementary Material 1 [file 12865_2024_621_MOESM1_ESM.docx]

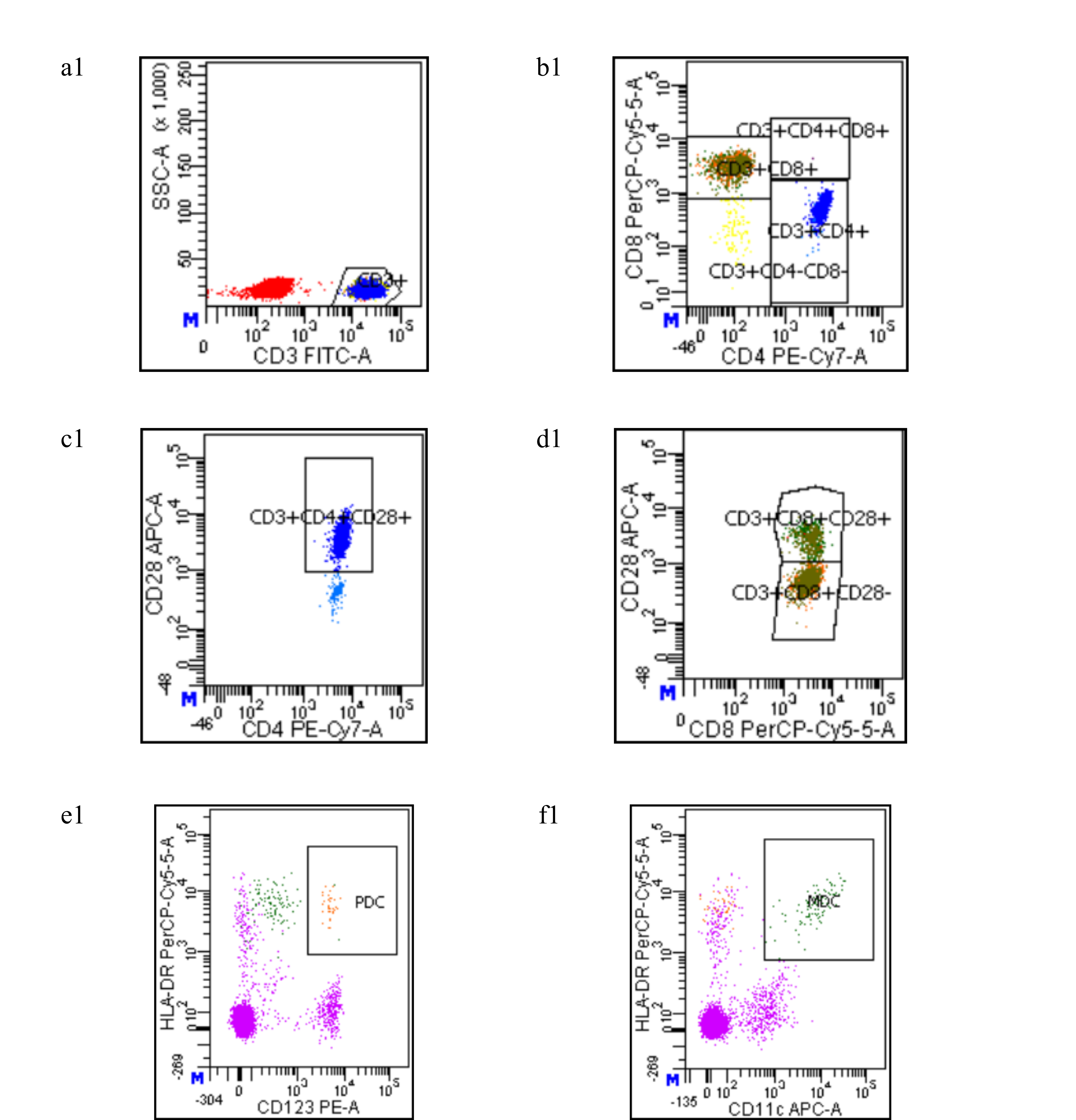


Supplementary figure1，Before treated CD3+, CD4+, CD8+, CD28+CD4+, CD28+CD8+, PDCs, MDCs, etc. in control group. a1 The flow cytometry image showed CD3+,b1 Showed CD4+,CD8+,c1 Showed CD28+CD4+,d1 Showed CD28+CD8+,e1 Showed PDCS,f1 Showed MDCs.


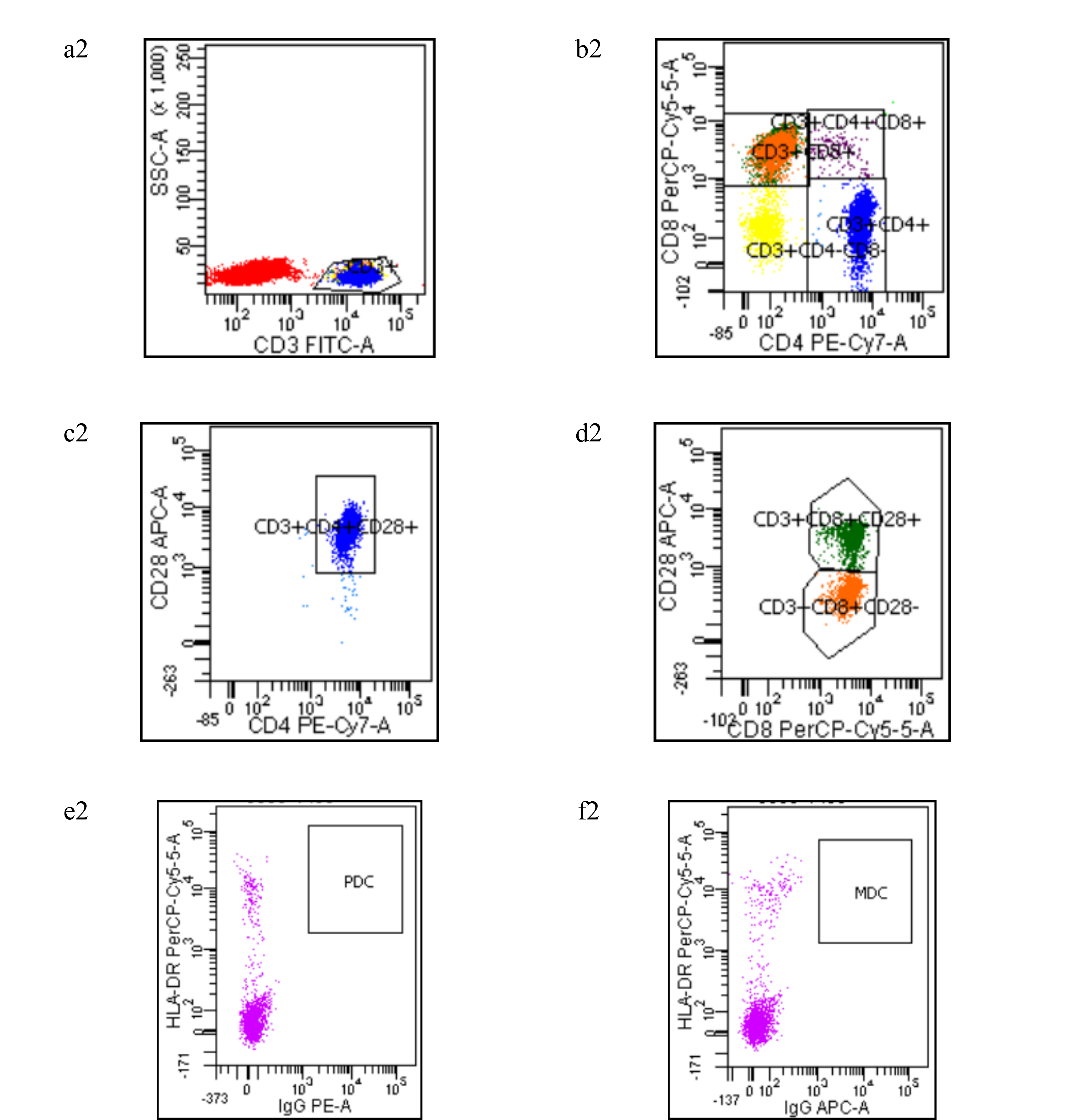


Supplementary figure2，After treated CD3+, CD4+, CD8+, CD28+CD4+, CD28+CD8+, PDCs, MDCs, etc. in control group. a2 The flow cytometry image showed CD3+,b2 Showed CD4+,CD8+,c2 Showed CD28+CD4+,d2 Showed CD28+CD8+,e2 Showed PDCS,f2 Showed MDCs.


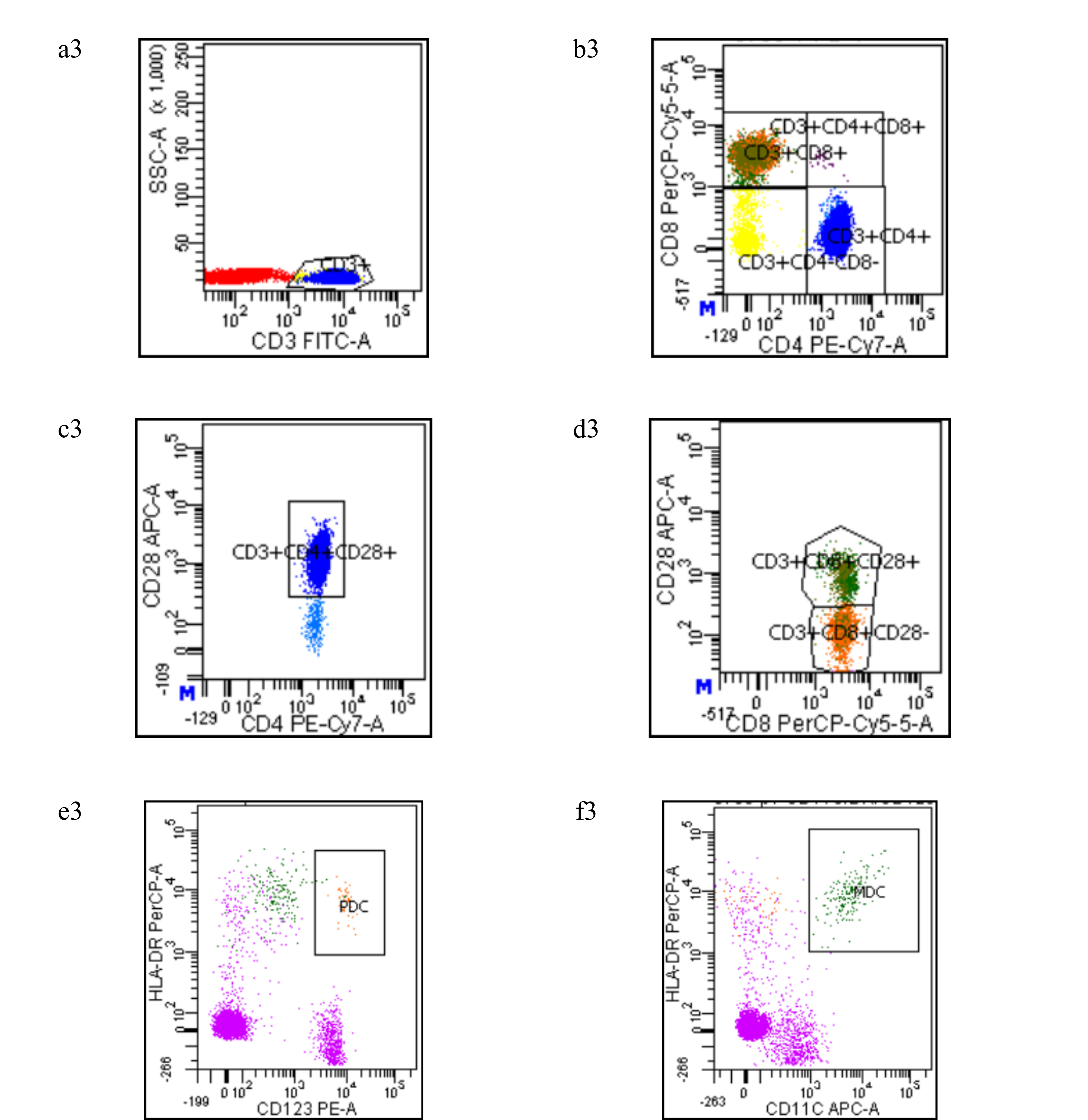


Supplementary figure3，Before treated CD3+, CD4+, CD8+, CD28+CD4+, CD28+CD8+, PDCs, MDCs, etc. in study group. a3 The flow cytometry image showed CD3+,b3 Showed CD4+,CD8+,c3 Showed CD28+CD4+,d3 Showed CD28+CD8+,e3 Showed PDCS,f3 Showed MDCs.


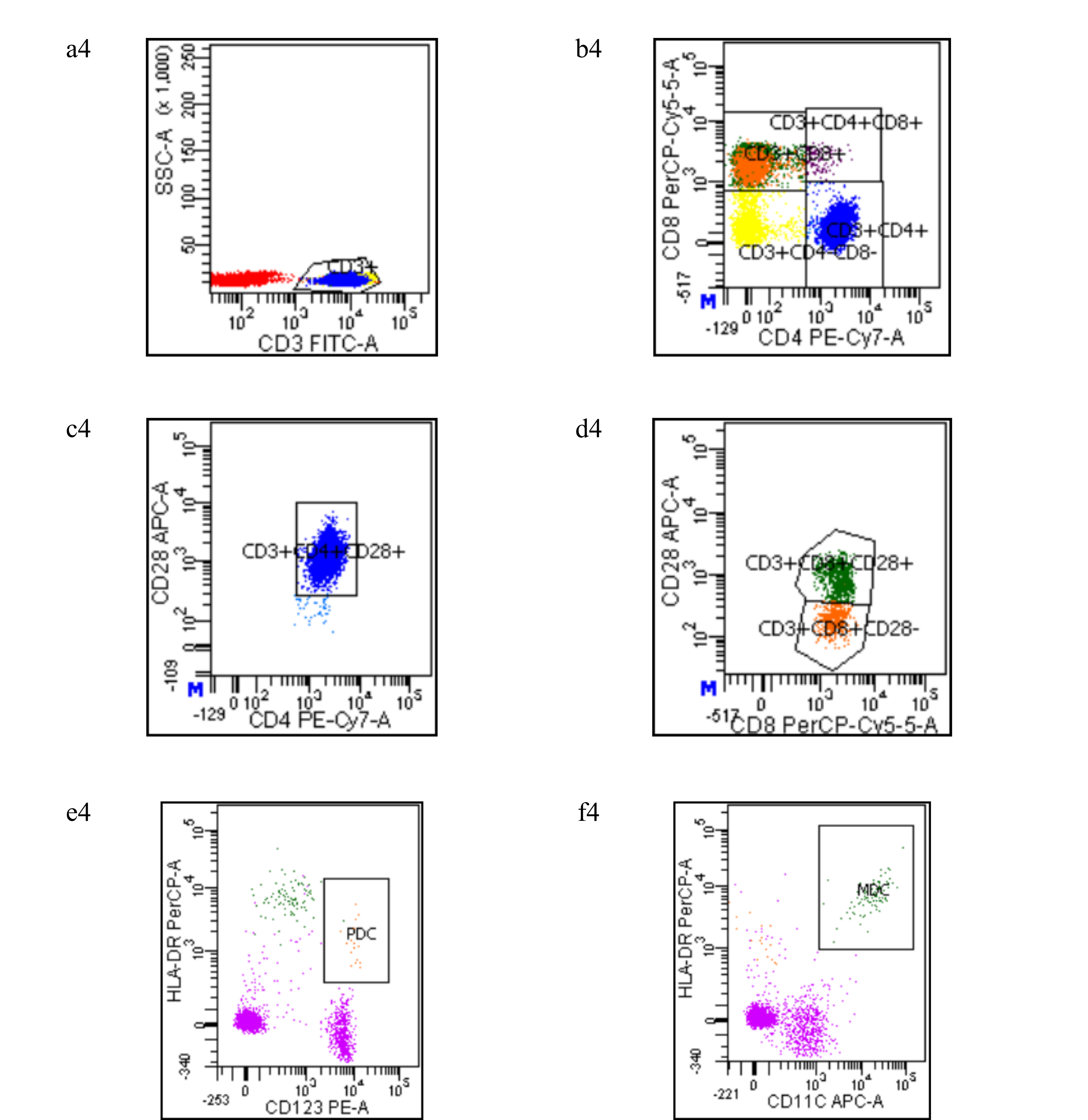


Supplementary figure4，After treated CD3+, CD4+, CD8+, CD28+CD4+, CD28+CD8+, PDCs, MDCs, etc. in study group. a4 The flow cytometry image showed CD3+,b4 Showed CD4+,CD8+,c4 Showed CD28+CD4+,d4 Showed CD28+CD8+,e4 Showed PDCS,f4 Showed MDCs.
