# Supplementary material for: Effect of immune-modulating metronomic capecitabine as an adjuvant therapy in locoregionally advanced nasopharyngeal carcinoma
Source: BMC Immunol. 2024 May 6;25:28. doi: 10.1186/s12865-024-00621-3 (PMC11071185; doi:10.1186/s12865-024-00621-3)
Supplement: Supplementary file 2 — Supplementary Material 2 [file 12865_2024_621_MOESM2_ESM.pdf]

Single-agent Capecitabine as Adjuvant chemotherapy in locoregionally advanced Nasopharyngeal carcinoma： a phase 3, randomised controlled trial

Protocol summary

Research	Adjuvant chemotherapy with capecitabine alone for locoregional advanced NPC
The applicant	Affiliated Cancer Hospital of Guizhou Medical University
Research	Randomized, open, controlled phase III clinical trial
Subject	Patients with locoregional advanced nasopharyngeal carcinoma
Number of	28 Cases

Enrollment criteria	1. Age is 18-65 years old<br>2. ECOG physical strength status score: 0~1 point.<br>**3. The initial diagnosis of viable radical radiotherapy is III-IVA (except T3-4N0,T3N1)** nasopharyngeal carcinoma patients (according to the 8th edition of the AJCC staging criteria), through the pathology histologically confirmed as non-keratotic carcinoma (according to the World Health Organization World Health Organization, Pathological classification of WHO).<br>4 Within 12 ± 2 weeks after completing the recommended radical radiotherapy.<br>5 No evidence of regional residual or distant metastasis was found before enrollment.<br>6 completed the recommended concurrent chemotherapy with ± induction chemotherapy.<br>7. Main organs have good function, that is, satisfied within 2 weeks before enrollment:<br>(1) Routine blood examination: hemoglobin> 90 g /L,Neutrophil $>1.5\times10^9$ /L; Platelet $>100\times10^9$ /L.<br>(2) Biochemical examination: total bilirubin ≤ 1.5×ULN (upper limit of normal value); Serum alanine aminotransferase (ALT) and aspartate aminotransferase (AST) ≤ 2.5×ULN; Alkaline phosphatase (ALP) ≤ 2.5×ULN; Creatinine clearance ≥ 50 ml/min.<br>8. Subjects voluntarily participated in this study, signed the informed consent form, with good compliance and cooperation Follow-up was conducted.

Exclusion criteria	1. Not known to tolerate capecitabine, or is allergic to its excipients.<br>2. There are multiple factors that affect oral medication (such as inability to swallow, chronic diarrhea, and Intestinal obstruction, etc.).<br>3 Previous history of malignancy, adequately treated basal cell carcinoma or squamous fine Except for cell carcinoma and cervical carcinoma in situ.<br>4. Women during pregnancy or lactation (pregnancy test should be considered for women of childbearing age Examination; emphasize effective contraception during treatment).<br>5 When receiving the recommended chemoradiotherapy, they received concurrent surgical treatment, or biological and exemption Epidemic treatment, etc.<br>6 Is receiving or is expected to receive other chemotherapy after receiving the recommended chemoradiotherapyDrug therapy, or biological and immunotherapy, etc.<br>7. Other radiation therapy before radical radiotherapy (if non-melanoma Skin cancer and previous lesions are located outside the target area of radiotherapy, except).<br>8 Have received chemotherapy or hand for the primary lesion and cervical metastases before receiving radical radiotherapy,surgical treatment (except for the diagnostic treatment).<br>9 Having other serious diseases may cause high risk or affect trial compliance nature. For example: unstable heart disease, kidney disease, chronic hepatitis, poor control of diabetes (fasting glucose> 1.5 ULN), and mental sickness.

Dosage regimen	Control group: follow-up observation;<br>Test group:single-agent capecitabine (650 mg/m bid, oral, d1-21, every 3 weeks). Patients were required to continue treatment until disease progression, unacceptable toxicity, or 1 year.Or take medicine for 1 year.
Statistical method	All efficacy analyses were performed on the basis of the intention-to-treat principle. All statistical tests were two-sided, and P values of less than 0.05 were considered.
Main study	Progression-free survival (PFS).
Secondary study	Overall survival (OS), distant metastasis-free survival (D-FFS), and no local regional recurrence inventory (LR-FFS), treatment toxicity, quality of life score (QoL).

Research background

Nasopharyngeal carcinoma (NPC) is significantly different from other head and neck cancers in terms of epidemiology, biological behavior, and treatment strategies.Nasopharyngeal carcinoma has significant regional and population clustering: its age-standardized incidence rate (per 100,000 male population) is 20-50 among indigenous people in Guangdong Province, China, and only 0.5 among predominantly white people. According to the International Agency for Research on Cancer (IARC), about 84,400 NPC patients were newly diagnosed worldwide in 2008, of which Chinese accounted for about 40%[1]. "Based on our previous data, 70% of NPC patients treated with IMRT or combined modality therapy from 2003 to 2006 were included.They belong to stage III or IV (seventh edition of AJCC staging criteria [2]), and they have a high rate of recurrence and metastasis [3]. In recent years, with the application of modern imaging and the progress of radiotherapy technology, the local control rate of NPC has been significantly improved, and distant metastasis has now become the main mode of treatment failure of NPC (FIG. 1)[4].

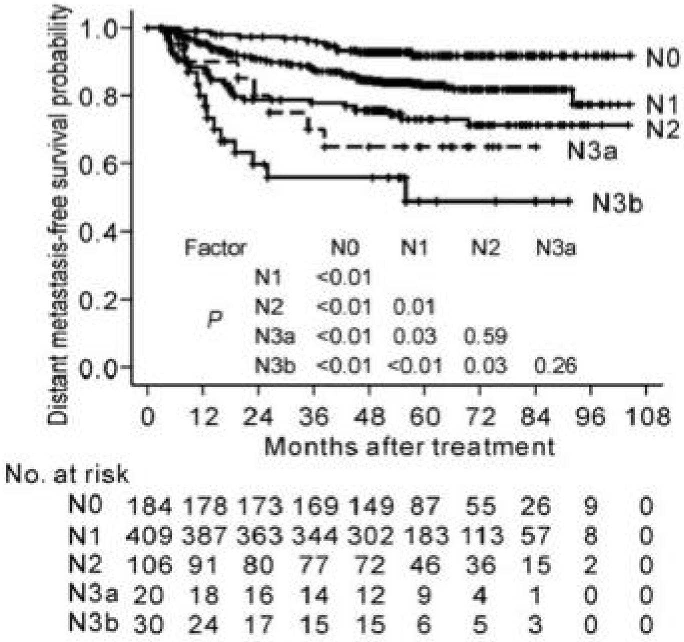

Figure 1 Rates of distant metastasis by N stage

In 2016, the National Comprehensive Cancer Network (NCCN) recommended concurrent chemoradiotherapy plus adjuvant/induction chemotherapy as the standard treatment for locoregionally advanced nasopharyngeal carcinoma. Multiple previous meta-analyses have shown that the survival benefit brought by chemotherapy on the basis of standard radiotherapy mainly comes from concurrent chemotherapy, while the significance of induction chemotherapy and adjuvant chemotherapy is not clear [5-7]. The newly published phase 3 clinical trial results showed that induction chemotherapy with docetaxel, cisplatin and 5-FU (TPF regimen) on the basis of concurrent chemoradiotherapy can effectively reduce the distant metastasis rate and improve the overall survival rate of patients with locoregionally advanced nasopharyngeal carcinoma (T3-4N0 patients with low risk of distant metastasis were excluded from this trial) [8]. In addition, gemcitabine combined with cisplatin (GP) regimen has relatively lower toxicity, and its superior efficacy has been confirmed in a recent phase 3 trial of advanced nasopharyngeal carcinoma [9,10]. Our ongoing study of GP induction chemotherapy in the locoregional region.The VALUE phase 3 trial in locally advanced nasopharyngeal carcinoma (NCT01872962) has completed enrollment, and the results are promising. According to the 2016 NCCN guidelines, TP or PF regimen is also a recommended induction chemotherapy regimen.

Compared with induction chemotherapy, the current recommended PF three-course adjuvant chemotherapy regimen for nasopharyngeal carcinoma has more toxic and side effects and poor compliance of patients. Our multicenter phase 3 clinical trial showed that PF adjuvant chemotherapy after concurrent chemoradiotherapy could not further improve survival, but only increased toxic and side effects [11]. It is a research hotspot to find an effective adjuvant chemotherapy regimen and beneficiary population to further improve the survival rate of patients with nasopharyngeal carcinoma. Lee et al. [12] conducted a combined analysis of two clinical trials of concurrent chemoradiotherapy followed by PF adjuvant

chemotherapy and found that the total dose of 5-FU in adjuvant chemotherapy was the only factor affecting distant metastasis of NPC. The higher the total dose, the better the distant metastasis control. In addition, a recent retrospective study by Liu et al. in Taiwan (Oral Oncol, Xiao Xiao) found that 1 year of adjuvant Tegafur-Uracil chemotherapy (ufudin) was efficacious in patients with high-risk locoregionally advanced nasopharyngeal carcinoma (high risk of distant metastasis) after definitive radiotherapy ± induction/concurrent chemotherapy. Its main components are tegafur and uracil, which can be converted into 5-FU in vivo) can effectively reduce distant metastasis and improve survival rate. The role of fluorouracil in adjuvant chemotherapy is worth exploring. In high-risk locoregionally advanced nasopharyngeal carcinoma, long-term oral fluorouracil antineoplastic drugs may improve patient compliance and provide survival benefits.

Capecitabine is a novel antimetabolic fluoropyrimidine deoxynucleoside carbamate agent that inhibits cell division and interferes with RNA and protein synthesis. After oral administration of capecitabine, it is converted into 5-Fu by thymidine phosphorylase (TP) in vivo. Since TP has higher activity in tumor tissues than in normal tissues, 5-Fu is "targeted" released in tumor tissues. Compared with 5-Fu, capecitabine has the characteristics of high efficiency and low toxicity. The literature reports that the effective rate of capecitabine monotherapy in the treatment of recurrent and metastatic nasopharyngeal carcinoma is 24-48% [13-15].The conventional regimen of capecitabine (1000-1250 mg/m2 bid, d1-14, q3w) was used in all patients. However, the side effects of this regimen are obvious, and the incidence of hand-foot syndrome in patients with recurrent and metastatic nasopharyngeal carcinoma is 23%-86%[13-15]. It is encouraging that the new regimen of continuous capecitabine until disease progression (650 mg/m2 bid, continuous, q3w) used in two phase 3 trials in breast and colorectal cancer is expected to reduce its side effects while preserving its efficacy [16,17]. Stockler et al. [16] compared a regimen of intermittent administration of capecitabine (1000 mg/m2 bid, d1-14, q3w) with capecitabine in 323 patients with advanced breast cancer.Regimen (650 mg/m2 bid, d1-21, q3w), cyclophosphamide, methotrexate and 5-FU were administered continuously.The efficacy and safety of the CMF protocol were evaluated. The results showed that compared with CMF regimen, capecitabine regimen improved overall survival, with lower toxicity and easier tolerance. Serious adverse effects were less common with continuous versus intermittent capecitabine (38% vs.47%), but efficacy was not significantly different. Simkens et al. [17] compared the efficacy of maintenance therapy with bevacizumab combined with continuous capecitabine (625 mg/m2 bid, d1-21, q3w) compared with observation alone in 558 patients with metastatic colorectal cancer, and found that the former significantly prolonged the progression-free survival time of metastatic colorectal cancer.

These results suggest the feasibility and potential efficacy of continuous administration of capecitabine in the adjuvant chemotherapy of locoregionally advanced nasopharyngeal carcinoma, which has a good application prospect. However, no clinical trial has investigated the efficacy of single-agent capecitabine

adjuvant chemotherapy in locoregionally advanced nasopharyngeal carcinoma. We therefore decided to conduct the first phase 3 multicenter, randomized, controlled clinical trial to determine the value of single-agent adjuvant capecitabine in the treatment of locoregionally advanced nasopharyngeal carcinoma. In this trial involving relatively high-risk patients with locoregionally advanced nasopharyngeal carcinoma (III-IVA,except T3-4N0, T3N1), we decided to use continuous single-agent capecitabine (650 mg/m2 bid, d1-21, q3w) on the basis of encouraging results from several previous clinical trials. The drug was continued until disease progression, intolerable toxicity, or for 1 year.

Objectives of the Study

Primary objective: To evaluate whether single-agent capecitabine adjuvant chemotherapy can improve progression-free survival (PFS) in patients with nasopharyngeal carcinoma (NPC) (III-IVA, except T3-4N0, T3N1) with a high risk of treatment failure.

Secondary objectives: overall survival (OS) and distant failure-free survival (distant failure-free survival) were compared between the two groups. D-FFS), locoregional failure-free survival (LR-FFS), capecitabine treatment toxicity and compliance, and quality of life score (QoL).

Study design

According to the specific conditions of the patients, after completing the recommended radical radiotherapy and the recommended concurrent chemotherapy ± induction chemotherapy, the patients who met the inclusion criteria within 12±2 weeks after the completion of radiotherapy were randomly divided into 2 groups according to the ratio of 1:1, namely the observation group (group 1) and the capecitabine group (group 2). In the capecitabine group, assessments were performed every 3 months after the initiation of the dose until 1 year after the initiation of the dose. For both the observation group and the capecitabine group, patients were to be followed at least every 3 months for the first 3 years after randomization, then every 6 months for years 3 through 5, and after 5 years, the review strategy was determined by the attending physician.

Treatment

The control group:follow-up observation.

Theexperimental group was adjuvant capecitabine.The regimen of capecitabine was 650 mg/m2 bid, orally, d1-21, every 3 weeks as a cycle. Protocol-prescribed medication, testing, and follow-up had to begin within 1 week after randomization and continued until disease progression, unacceptable toxicity, or 1 year.

Statistical Analysis

According to the nature of the analysis indicators, the analysis methods of statistical description and statistical test were determined. The distribution range, mean, median, standard deviation and interquartile range IQR of continuous variables were analyzed, and the two groups were compared by t test or nonparametric test. The number of cases and incidence were analyzed by the list of categorical variables. Chi-square test, fisher's exact probability method or contingency table data statistical analysis method were used to compare the categorical variables between the two groups. All statistical tests were two-sided, and a P value of 0.05 or less was considered to indicate statistical significance.
